# A prediction model for distinguishing lung squamous cell carcinoma from adenocarcinoma

**DOI:** 10.18632/oncotarget.17038

**Published:** 2017-04-11

**Authors:** Hui Li, Zhengran Jiang, Qixin Leng, Fan Bai, Juan Wang, Xiaosong Ding, Yuehong Li, Xianghong Zhang, HongBin Fang, Harris G Yfantis, Lingxiao Xing, Feng Jiang

**Affiliations:** ^1^ Department of Pathology, Hebei Medical University, Shijiazhuang, Hebei, China; ^2^ Department of Pathology, the University of Maryland School of Medicine, Baltimore, Maryland, USA; ^3^ The F. Edward Hébert School of Medicine at the Uniformed Services University of the Health Sciences, Bethesda, Maryland, USA; ^4^ Department of Pathology, Second Hospital of Hebei Medical University, Shijiazhuang, Hebei, China; ^5^ Department of Biostatistics, Bioinformatics and Biomathematics, Georgetown University Medical Center, Washington, D.C., USA; ^6^ Pathology and Laboratory Medicine, Baltimore Veterans Affairs Medical Center, Baltimore, Maryland, USA

**Keywords:** MiRNA, biomarkers, lung cancer, histology, cytology

## Abstract

Accurate classification of squamous cell carcinoma (SCC) from adenocarcinoma (AC) of non–small cell lung cancer (NSCLC) can lead to personalized treatments of lung cancer. We aimed to develop a miRNA-based prediction model for differentiating SCC from AC in surgical resected tissues and bronchoalveolar lavage (BAL) samples. Expression levels of seven histological subtype-associated miRNAs were determined in 128 snap-frozen surgical lung tumor specimens by using reverse transcription-polymerase chain reaction (RT-PCR) to develop an optimal panel of miRNAs for acutely distinguishing SCC from AC. The biomarkers were validated in an independent cohort of 112 FFPE lung tumor tissues, and a cohort of 127 BAL specimens by using droplet digital PCR for differentiating SCC from AC. A prediction model with two miRNAs (miRs-205-5p and 944) was developed that had 0.988 area under the curve (AUC) with 96.55% sensitivity and 96.43% specificity for differentiating SCC from AC in frozen tissues, and 0.997 AUC with 96.43% sensitivity and 96.43% specificity in FFPE specimens. The diagnostic performance of the prediction model was reproducibly validated in BAL specimens for distinguishing SCC from AC with a higher accuracy compared with cytology (95.69 vs. 68.10%; *P* < 0.05). The prediction model might have a clinical value for accurately discriminating SCC from AC in both surgical lung tumor tissues and liquid cytological specimens.

## INTRODUCTION

More than 85% of lung cancers are non-small cell lung cancer (NSCLC), which is a smoking-related disease and the leading cancer killer in the USA and worldwide. NSCLC mainly consists of two major histological subtypes: squamous cell carcinoma (SCC) and adenocarcinoma (AC) [1]. Since chemotherapy and radiation therapy of NSCLC differ according to the subtypes, accurately differentiating SCC from AC is clinically important for the personalized treatment of the malignancy [2]. For instance, although AC is more susceptible to the antifolate drug pemetrexed, SCC is largely unresponsive to it [3]. SCC patients treated with the anti-angiogenic agent bevacizumab may suffer from pulmonary hemorrhage [4]. NSCLC patients with SCC have a higher rate of local failure after stereotactic body radiation therapy compared with patients with AC [5]. Furthermore, as new immunotherapy enters the clinical arena, precisely distinguishing SCC from AC becomes even more important. For instance, among patients with advanced non-SCCs that have progressed during or after platinum-based chemotherapy, overall survival would be longer with nivolumab that blocks the programmed cell death protein 1 (PD-1) immune checkpoint pathway than with docetaxel, a chemotherapy medication [6].

The standard histopathologic methods, including analysis of morphology and protein expression in surgical and small biopsy specimens are used for identifying AC and SCC subtypes [2]. However, these current methods may produce incorrect diagnosis in 15%–35% cases [7, 8]. Furthermore, surgical lung tissue and biopsy specimens are invasively obtained, which are not suitably for preoperative subclassification of NSCLC. Cytological approaches are commonly used for collecting specimens in the preoperative setting, due to the ease of sample collection and minimal trauma to the patients [9–11]. Cytological materials mainly include fine-needle aspiration (FNA), bronchial brushing, and bronchoalveolar lavage (BAL). FNA provides better accuracy than do bronchial brushing and BAL samples. However, procedure-related complications and mortality are higher for FNAs compared with bronchial brushings and BALs [12, 13]. Bronchial brushings and BALs are liquid cytological specimens, and may provide appropriate materials for the minimal invasive diagnosis of lung cancer, particularly, when biopsies and FNAs are not concurrently acquired [10]. Among these liquid specimens, bronchial brushings might provide high-percentage identification for malignant cells compared with BALs. However, since bronchial brushings are taken from bronchial lesions through catheter-based brushing under direct visualization, they are not available if no visible lesions can be targeted. By contrast, BALs could be sampled from a larger area of the bronchial mucosa, enabling a more satisfactory specimen that could not be collected by bronchial brushing [14]. Therefore, examination of BAL specimens provides an approach for preoperative diagnosis of lung cancer, especially, in many cases BAL samples are the only available materials [9]. However, cytological study of BALs for accurate subclassification of NSCLC might be difficult, because of the absence of the determining tissue architectures, paucity of tumor cells, poor cell differentiation, and interobserver variability. Instead of observing morphologic characterization by cytology, molecular study of BALs could identify tumor-related molecular aberrations in the specimens, which are specific signs of lung cancer cells. Therefore, molecular biomarkers that can be detected in BAL samples for discriminating SCC from AC are urgently needed.

MicroRNA (miRNA) is a small non-coding RNA molecule (containing about 22 nucleotides) and functions in RNA silencing and post-transcriptional regulation of gene expression [15]. Dysregulation of miRNAs plays a crucial role in lung tumorigenesis [16]. Furthermore, miRNA are stably preserved and reducibly measurable in a variety of clinical specimens [17]. Analysis of lung tumor-associated miRNAs provides a potential approach for lung cancer diagnosis and classification of the histological types [16, 18–21]. For example, we have shown that determination of miRNA expression in sputum could diagnose lung cancer at the early stage [22–29]. Furthermore, using a reverse transcription-PCR (RT-PCR)-based assay, we have developed a panel of three sputum miRNA biomarkers, including miR-205, for specifically diagnosing lung SCC [28]. Lebanony et al. demonstrated that analysis of a single miRNA, miR-205, could classify AC and SCC subtypes in surgical and biopsy tissues specimens with accuracy rates of 90%–100% [30, 31]. Patnaik et al further showed that combined use of miR-375 and miR-205 could subtype NSCLC in paraffin-embedded formalin-fixed (FFPE) biopsies and resectates with an accuracy of 96% [2]. In addition, Hamamoto et al identified another set of miRNAs consisting of miRs-196b, 205 and 375 for distinguishing SCC from AC in surgical lung tumor tissues with 76% sensitivity and 80% specificity [32]. The previous studies demonstrated that miRNAs provided potential classifiers for discriminating SCC from AC. Furthermore, miR-205 has been consistently reported as a useful biomarker for distinguishing SCC from AC. Nevertheless, two challenges remain. 1), there is an argument about which miRNAs could be used in combination with miR-205 to provide a high accuracy for reproducibly distinguishing SCC from AC, given that a single biomarker could not provide sufficient diagnostic power. 2), although the miRNAs have been tested in FNAs and bronchial brushings, the diagnostic potential has not been investigated in BALs, which, in many cases, are the only available materials for the diagnosis and subclassification of NSCLC [9].

Using next-generation deep sequencing (NGS) to comprehensively characterize and compare miRNA profiles of surgically resected SCC and AC tissues [33], we recently identified four miRNAs (miRs-944, 205-5p, 135a-5p, and 577) that exhibited distinctive expression levels in SCC versus AC. On the basis of our and others’ earlier findings [28, 33, 2, 9, 10, 31, 32, 34], here we aimed to develop miRNA biomarkers for accurately and reproducibly differentiating SCC from AC in surgical tissues and BAL samples.

## RESULTS

### Patients and specimens

The study protocols were approved by the Institutional Review Boards of the University of Maryland Medical Center (UMMC; Baltimore, MD, USA) and Hebei Medical University (Shijiazhuang, China). This study consisted of three phases: developmental, validation, and application phases. 1), the developmental phase was to identify and develop a miRNA-based prediction model for distinguishing between SCC and AC in surgical resected specimens. Surgical tissues were obtained from 128 lung cancer patients who had either a lobectomy or a pneumonectomy between March 1, 2013 and September 25, 2015 at the UMMC. All cases were diagnosed with histologically confirmed NSCLC, consisting of 62 SCCs and 66 ACs. Furthermore, manual macrodissection was performed on the tissues as described in our previous study [35] to ensure the presence of more than 85% cancer cells in each sample. Demographic and histopathological characteristics of the NSCLC patients and tumors are shown in Tables 1, 2), the validation phase was to confirm the prediction model for the subclassification of NSCLC in a different set of lung tumor tissue specimens. To this end, formalin-fixed paraffin-embedded (FFPE) specimens were prepared from the surgical tissues of 112 lung cancer patients who had either a lobectomy or a pneumonectomy at Hebei Medical University. The FFPE lung tumor tissues consisted of 55 SCCs and 57 ACs (Tables 1, 3), the application phase was to determine if the prediction model could be useful in bronchoscopically collected BAL samples for distinguishing SCC from AC. We prospectively recruited patients who underwent bronchoscopy because of suspicious clinical or radiological findings at the UMMC. The selection of BAL samples for this study was undertaken on the basis of an adequate cytology preparation containing endobronchial cells and alveolar macrophages, and metaplastic or tumor cells [36]. One hundred and twenty seven specimens met these criteria and thus were used in this project. Of the 127 NSCLC patients with BAL samples, 82 were finally diagnosed to have SCC, while 45 have AC of lung cancer (Table 1). One half of the resulting BAL fluid was immediately centrifuged at 1,000xg for 15 min for routine cytological assessments. Other half of the BAL fluid was washed in phosphate buffered saline (Sigma-Aldrich, St. Louis, MO), from which cell pellets were prepared and stored at −80°C until being tested for molecular analysis.

**Table 1:** Demographic and histopathological characteristics of NSCLC patients and specimens

	Developmental phase (128 frozen tumor tissues)	Validation phase (112 formalin-fixed, paraffin-embedded tumor tissues)	Application phase (127 bronchoalveolar lavages)
SCC	62	57	82
Age, year (mean ± SD)	67 ± 9	61 ± 8	66 ± 8
Sex			
Male	39	42	53
Female	23	15	29
TNM stage			
I	25	28	34
II	18	15	25
III-IV	19	14	23
AC	66	55	45
Age, year (mean ± SD)	68 ± 10	61 ± 8	66 ± 8
Sex			
Male	43	27	29
Female	23	26	16
TNM stage			
I	26	31	18
II	20	10	13
III-IV	20	14	14

Abbreviations: NSCLC, non-small cell lung cancer. SCC, squamous cell carcinoma; AC, adenocarcinoma; TNM, tumor, node, metastasis classification.

**Table 2:** Expression of seven miRNAs in SCC vs. AC tissues of the developmental phase

miRNAs	Mean ± SD in SCC tissues	Mean ± SD in AC tissues	*P*	AUC ± SD
miR-205-5p	3.3890 ± 0.6516	0.1216 ± 0.0252	< 0.0001	0.9562 ± 0.0115
miR-944	0.0031 ± 0.0011	0.0001 ± 2.871e-005	< 0.0001	0.9482 ± 0.0240
miR-34a	5.9053 ± 2.3547	3.2238 ± 1.3036	< 0.0001	0.7649 ± 0.0357
miR-135a-5p	1.2379 ± 0.7716	2.2507 ± 1.0837	0.0013	0.7267 ± 0.0522
miR-375	0.1268 ± 0.0638	0.2648 ± 0.0922	0.0010	0.7206 ± 0.0588
miR-577	0.0013 ± 0.0003	0.0003± 6.871e-005	0.0269	0.6612 ± 0.0549
miR-21-5p	6.2587 ± 1.0835	6.0329± 1.0128	0.8145	0.5027 ± 0.0043

Abbreviations: NSCLC, non-small cell lung cancer; SCC, squamous cell carcinoma; AC, adenocarcinoma; SD, standard deviation.

**Table 3:** Comparison of a panel of miRNA biomarkers and cytology for distinguishing lung SCC from AC in 58 BAL samples*

	Accuracy (95% CI)	Sensitivity (95% CI)	Specificity (95% CI)
A prediction model	95.69% (86.27% to 98.65%)	94.83% (85.62% to 98.92%)	96.55% (85.62% to 98.92%)
Cytology	68.10% (56.39% to 78.62%)	77.59% (64.73% to 87.49%)	58.62% (44.93% to 71.40%)

Abbreviations: BAL, SCC, squamous cell carcinoma; AC, adenocarcinoma; bronchoalveolar lavage. *, All *p* values < 0.05.

### Developing a miRNA-based prediction model for distinguishing SCC from AC in surgical tumor tissue specimens

**Figure 1:**
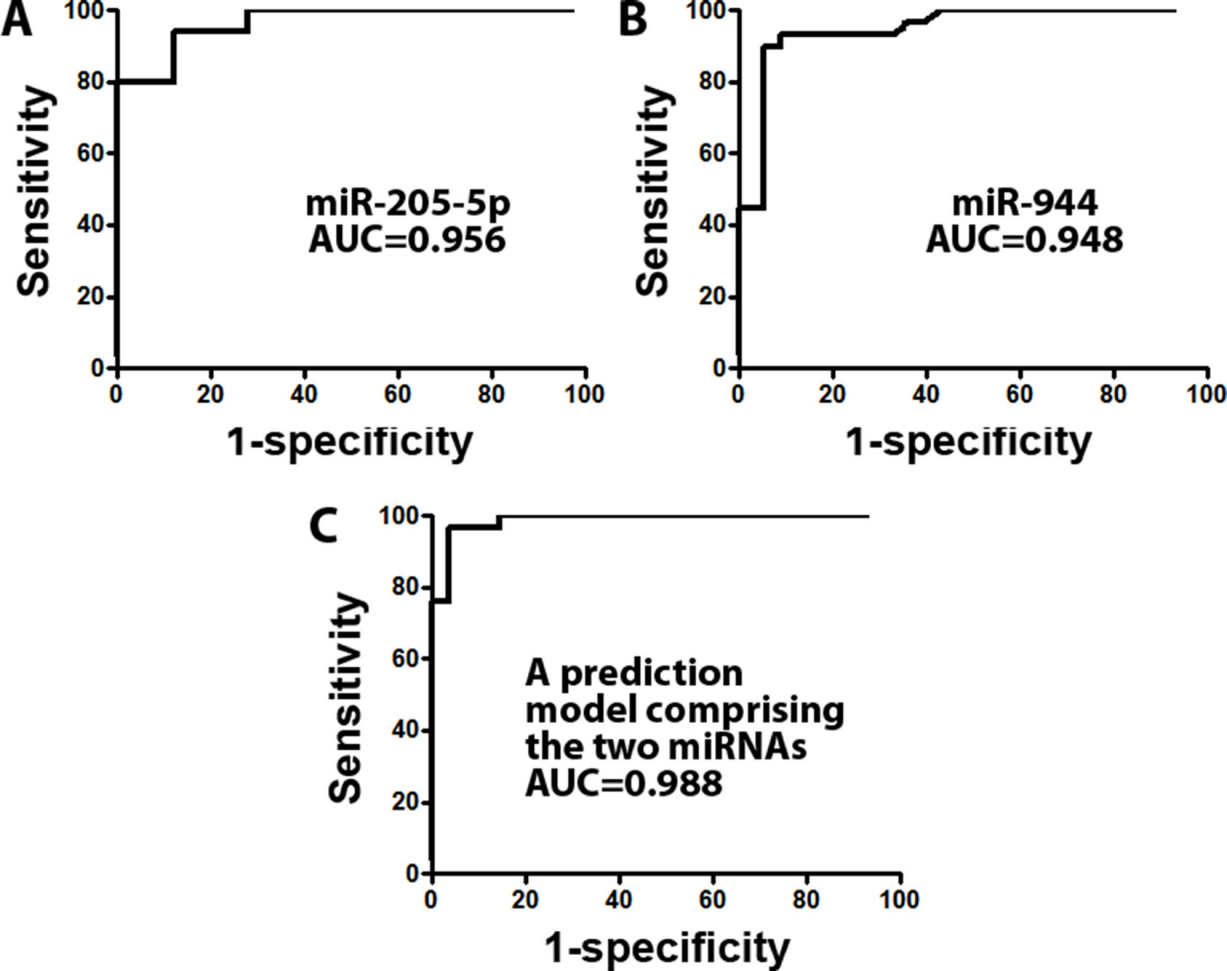
A prediction model based on two miRNAs (miRs-205-5p and 944) was developed for distinguishing SCC from AC in frozen lung tumor tissues (**A**) the receiver operating characteristic (ROC) curve of miR-205-5p produced an area under the ROC curve (AUC) of 0.956. (**B**) miR-944 created an AUC of 0.948. (**C**) a prediction model with the two miRNAs produced AUC of 0.988 for differentiating SCC from AC.

Four miRNAs (miRs-944, 205-5p, 135a-5p, and 577) identified in our previous study [33] and three miRNAs (mRs-21, 34a, and 375) identified by others [2, 9, 10] whose changes were specifically associated with SCC were included in this study. Reverse transcription polymerase chain reaction (RT-PCR) showed that all seven miRNAs (miRs-21, 34a, 135a-5p, 205-5p, 375, 577, and 944,) and U6 had ≤ 30 Ct (cycle threshold) values in each tissue sample. Yet no product was synthesized in the negative control samples. Therefore, the genes were reliably detectable in the tissue specimens by using RT-PCR assay. Of the seven miRNAs, six (miRs-944, 205-5p, 135a-5p, and 577, 34a, and 375) displayed a significantly different level in SCC versus AC tumors (all *P* < 0.05) (Table 2). The expression levels of miRs-34a, 205-5p, 577, and 944 were higher in SCC compared with AC specimens (All *P* < 0.05) (Table 2). Conversely, a lower expression level of miRs-135a-5p and 375 was observed in SCC compared with AC samples (All *P* < 0.05) (Table 2). Furthermore, the individual miRNAs exhibited AUC values of 0.6612-0.9562 for the classification of the two types of NSCLC (Table 2). From the six miRNA candidates, we used stepwise logistic regression models with backward model selection to construct a logit model for discriminating SCC from AC. Probability for differentiating SCC from AC = eU/(1+eU), where e is the base of the natural logarithm, U = 2.6389 + 1.2662 × log (miR-205-5p) + 0.3269 × log (miR-944). miRs-205-5p and 944 were selected in the model, which had an AUC of 0.988 for distinguishing SCC from AC tumors (Figure 1). The cut-off value for the model was set at 2.568 by using the highest Youden Index [37]. Moreover, including other miRNAs (mRs-21, 34a, 135a-5p, 375, and 577) in the model did not improve the efficiency for subtyping NSCLC. Subsequently, the use of miRs-205-5p and 944 in combination generated an accuracy of 96.48% with 96.55% sensitivity and 96.43% specificity for differentiating SCC from AC. The two miRNAs had no statistically significant association (All *p* > 0.05) with stages of the NSCLC cases, and the age, gender, and ethnicity of the patients.

### Validating the prediction model in an external cohort of FFPE specimens

**Figure 2:**
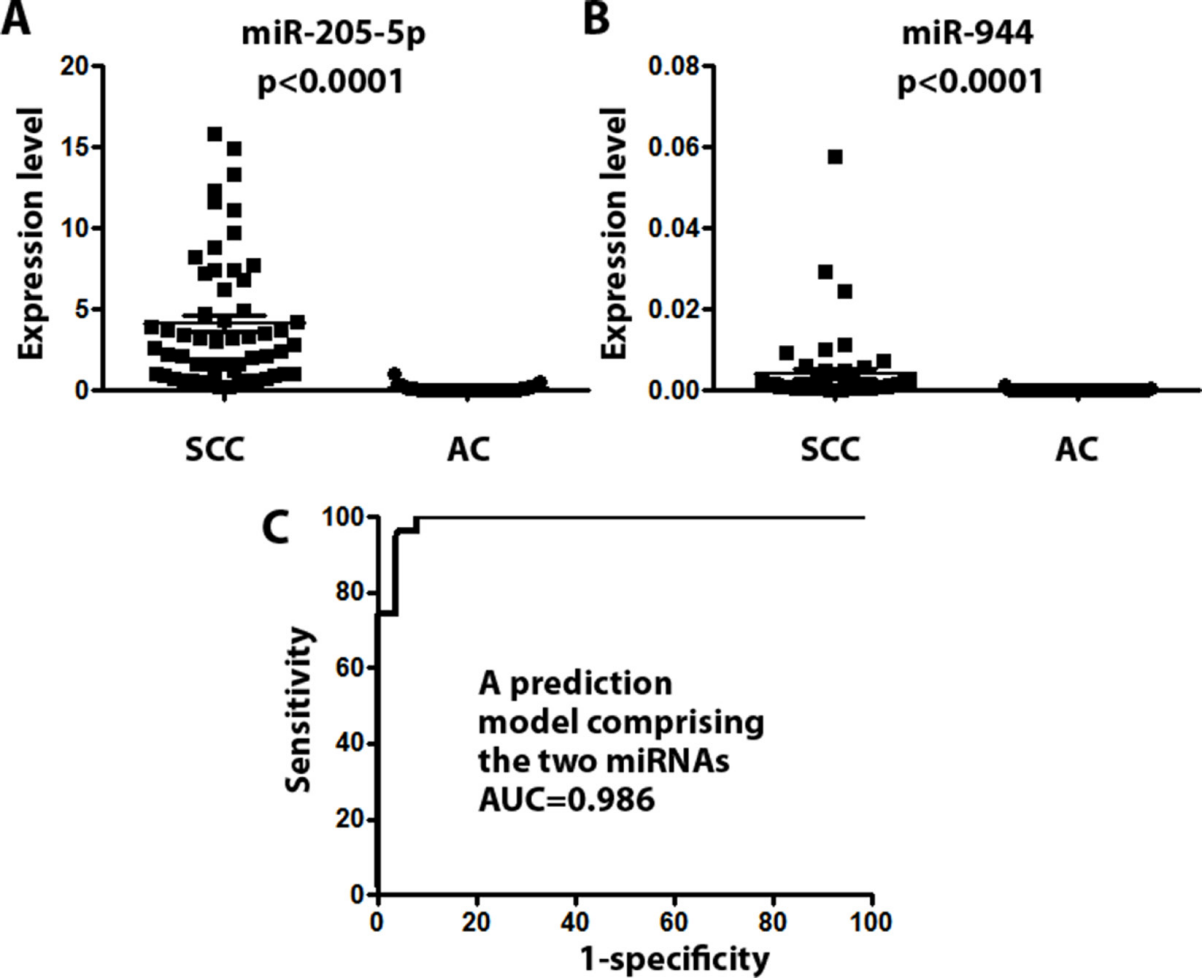
The diagnostic performance of the prediction model with two miRNAs (miRs-205-5p and 944) for the discrimination of SCC from AC was successfully validated in formalin-fixed, paraffin-embedded lung tumor tissues collected in geographically distant populations (**A**) miR-205-5p displayed a significantly higher level in SCC compared with AC specimens (*P* < 0.0001). (**B**) miR-944 exhibited a significantly higher level in SCC compared with AC specimens (*P* < 0.0001). (**C**) the prediction model consisting of the two miRNAs produced AUC of 0.986 for differentiating SCC from AC.

The two miRNAs defined from the above developmental phase were validated in an independent cohort of 112 FFPE specimens consisting of 57 SCC and 55 AC tissues collected in China. Similar expression profiles of the two miRNAs in SCC and AC tumors were observed in the validation phase as did in the developmental phase: The expression levels of miRs-205-5p and 944 were significantly higher (All *p* < 0.05) in SCC compared with AC specimens (Figure 2). Applying the prediction model consisting of the two miRNAs in the FFPE samples produced an AUC of 0.986 in discriminating SCC from AC (Figure 2). As a result, the prediction model created 96.43% accuracy with 96.43% sensitivity and 96.43% specificity, therefore confirming the ability for discriminating SCC from AC.

### Applying the prediction model in BAL specimens for differentiating SCC from AC of NSCLC

RT-PCR analysis of the two miRNAs (miRs-205-5p and 944) and U6 was performed in BAL samples of 127 patients who underwent bronchoscopy by using the same RT-PCR protocol as described above. miR-205-5p had a Ct value of more than 35 in 64% of the 127 BAL samples. Furthermore, miR-944 and U6 had a Ct value of more than 35 in all the 127 BAL samples. Therefore, amplification curves of the RT-PCR analysis for the miRNAs and U6 were not reliably generated, implying that expression of the genes in the BALs might be too low to be detectable by RT-PCR. We have previously demonstrated that Droplet Digital PCR (ddPCR) is more sensitive with greater precision and reproducibility to detect expression level of miRNAs than does the conventional RT-PCR assay [38, 39]. Furthermore, ddPCR needs much less RNA compared with RT-PCR, and is particularly useful in the quantification of the miRNAs that have endogenous low-level expression in clinical samples. We, therefore, used ddPCR to determine expression level of the two miRNAs and U6 in the BAL samples. Each well of the samples contained at least 10,000 droplets. By contrast, no product was synthesized in the negative control samples. Thus, the samples were successfully ‘‘read’’ for the absolute quantification of the two miRNAs by using a cost-effective, reliable and accurate assay in the 127 BAL specimens. In addition, the prediction model with the two miRNAs generated an AUC of 0.997 for the discrimination of SCC from AC. Consequently, the miRNA biomarker panel had 96.12% accuracy with 95.00% sensitivity and 96.83% specificity for classification of SCC from AC. Cytological study was also done in the 127 BAL samples. However, due to disproportionate amount of bronchial epitheliums that had poor cellar morphology, cytology was successfully performed on only 58 of the 127 BAL samples. Our observation of a low diagnostic rate of cytology in BALs was consistent with the previous findings by others [40]. Therefore, cytological analysis of the BALs had a lower diagnostic rate (45.6%) compared with the prediction model (100%) (*P* = 0.01). Of the 58 NSCLC cases that had cytological diagnosis in BALs, 39 were SCC and 19 were AC on the basis of histological diagnosis of bronchoscopic biopsies or surgical specimens. In the 58 BAL samples, cytology had 68.10% accuracy with 77.59% sensitivity and 58.62% specificity, while the prediction model created 95.69% accuracy with 94.83% sensitivity and 96.55% specificity for differentiating SCC from AC (Table 3). The biomarker-based prediction model had higher sensitivity and specificity compared with cytology in discriminating SCC from AC (All *P* < 0.05). However, the addition of the cytology study in the biomarker panel did not improve the diagnostic efficiency for distinguishing SCC from AC in the BAL samples. Therefore, the prediction model not only subtypes histology of the BAL samples that are not able to be analyzed by cytology, but has a higher accuracy for discriminating SCC from AC compared with cytology.

## DISCUSSION

Previous studies including our own work have shown that miR-205 is expressed exclusively in SCC tissues, and hence acts as a specific biomarker for SCC type of NSCLC [2, 10, 27, 31, 33, 34, 41–43]. Analysis of miR-205 in surgical tissues, biopsy, and bronchial brushings specimens could distinguish SCC from AC [2, 10, 31, 34, 41, 42]. Yet a single biomarker could not provide sufficient diagnostic value. For instance, using miR-205 as a biomarker had 96% sensitivity, whereas only 90% specificity for subtyping between SCC and AC [34]. Therefore, efforts have bene made to identify additional miRNAs, such as miRs-21, 34a, and 375, that could be used together with miR-205 to have higher accuracy and reproducibility compared with miR-205-5p alone [2, 10, 31, 34, 41, 42]. However, these recently identified miRNAs also exhibited abnormal expressions in AC tissues compared with normal lung tissues, and therefore might not be SCC-specific molecular aberrations. As a result, combined use of these miRNAs with miR-205 generated diverse panels of biomarkers, which, however, produced widespread inconsistent results. Using whole genomic NGS analysis to systematically and comprehensively define and compare miRNA profiles in SCC and AC tissues [33], we identified SCC-specific miRNA aberrations, including miRs-205-5p and 944, whose elevated expression was restricted to SCC [33]. This present study using three large and independent sets of specimens confirms that the high expression level of miR-205-5p and 944 is restrained in SCC tissues. Furthermore, a simple prediction model consisting of the two miRNAs is developed as a classifier for specifically distinguishing SSC from AC. The simple equation has maximizes sensitivity and specificity with a single cut-off value. Compared with the previous complex score system for analysis of multiple and different variables, the friendly used equation could provide a more convenient approach for the classification of NSCLC types. In addition, the performance of the small panel of miRNAs was reproducibly validated in an independent set of FFPE tissues collected in geographically distant populations in China, further implying the usefulness of the approach for distinguishing SCC from AC.

Many patients with NSCLCs present at a stage when their tumors are surgically unresectable [44]. The only clinical materials from these patients are usually small cytological specimens, including transbronchial FNA biopsy, bronchial brushings, and BALs. Therefore, a molecular assay that can accurately classify histological types of NSCLC using the cytological samples is clinically important. A panel of two miRNAs consisting of miRs-205 and miR-375 was developed that could be used in FNA biopsy specimens for differentiating SCC from AC [2]. Furthermore, a different panel of two miRNAs (miRs-205 and miR-34a) was developed that could be used in bronchial brushing specimens [10]. However, many peripheral lung lesions can't be reached by a bronchoscope, by which FNA biopsy and bronchial brushing specimens are not collectable. Since BALs are obtained by instilling saline into a tumor bearing segment and retrieving samples, the specimens are near ideal materials for cytological diagnosis of the peripherally located lung tumors when FNA and bronchial brushing specimens are not available. However, there are two major obstacles in the use of BALs for lung cancer diagnosis and subclassification. 1), in cases with poorly differentiated tumors or distorted BAL specimens, the morphological observation for subtyping NSCLC is not always possible on the basis of cytology alone [45]. For instance, Kawaraya et al collected BAL samples from 81 patients with lung cancer having no visible endoscopic findings. Of the 81 samples, cytology was successfully done in only 28 BAL samples, producing a diagnostic rate of 34.6% [40]. Rather than observing morphologic characterization, molecular study of BALs could identify tumor-related miRNA aberrations in BALs, thus provide a more efficient tool. Our present study for the first time reports the importance of miRNA profiles in BALs for discriminating between SCC and AC. 2), due to the low concentration of RNA typically obtained from the cytological samples and the low abundance of the miRNAs (e.g., miR-944 and U6) of interest, measuring the miRNAs by using conventional RT-PCR in BAL samples is especially difficult. We have demonstrated that ddPCR is 100 times more sensitive with greater precision and reproducibility to quantify miRNAs compared with RT-PCR [38, 39]. Furthermore, ddPCR needs much less (approximately 100 times less) RNA compared with RT-PCR. Therefore, ddPCR is particularly useful in determining expression of the miRNAs that have endogenous low expression in the liquid cytological samples. Indeed, our current study demonstrates that the two miRNAs and U6 in BALs could reproducibly be determined in BALs by ddPCR rather than conventional RT-PCR. Furthermore, limited tumor cell sampling in BALs does not diminish the discriminating power of the miRNA biomarkers. In addition, ddPCR analysis of the two miRNAs showed a similar diagnostic performance in BAL samples as in tissue specimens. Moreover, the miRNA panel using ddPCR not only analyzes the BALs for which cytology has failed at diagnosing or subclassifying NSCLC due to disproportionate amount of bronchial airway material (epithelial cells), but also has a higher accuracy for discriminating SCC from AC compared with cytology. Therefore, the miRNA biomarker-based prediction model may overcome the obstacles in using BAL samples for lung cancer diagnosis and classification.

Elevated miR-205 expression was suggested to participate in the development and progression of lung SCC [33, 34]. We have shown that miR-205 is one of three miRNAs that could be used as sputum biomarkers for the early detection of lung SCC [28]. In the miR-205 family, there are two members (miRs-205-3p and 205-5p), which may have different biological functions in lung tumorigenesis. Our deep sequencing analysis identified miR-205-5p rather than miR-205-3p as a unique signature for SCC [33]. This present study further suggests that the elevated miR-205-5p expression could be a specific sign of the subtype of SCC. Like miR-205-5p, miR-944 is highly expressed in lung SCC but not in AC [33]. We have confirmed that miR-944 has oncogenic function in pathogenesis of SCC, since its dysregulation contributes to cancer cell growth, proliferation and invasion by targeting SOCS4, a tumor suppressor [33]. Interestingly, miR-944 is located in the intron of the tumor suppressor *p63*, while antibody for P63 protein is an immunohistochemistry marker for lung SCC [46]. Here we demonstrate that a high expression of miR-944 is uniquely restricted to SCC, and its combined use with miR-205-5p produces a higher accuracy in distinguishing between SCC and AC compared with either miR-205-5p or miR-944 used alone. However, direct comparison of immunohistological analysis of P63 and ddPCR quantification of miR-944 in the same set of specimens for the discriminating SCC and AC is required.

In summary, compared with previous reports on miRNA expression in subclassifying NSCLC, our study is unique for the following reasons: First, from the SCC-specific miRNA biomarker candidates, we develop a simple prediction model that can accurately and reproducibly distinguish SCC from AC. Second, our study might be the first to report the importance of miRNA profiles in BALs for discriminating between the two major histological entities of NSCLC. The prediction model would have the clinical potential in optimizing treatment strategies based on lung cancer subtypes. Nevertheless, a large and prospective study for comprehensively validating the biomarkers for discriminating SCC from AC in multiple centers is needed.

## MATERIALS AND METHODS

### Patients, specimens, and study design

This study consisted of three phases: developmental, validation, and application phases. 1), the developmental phase was to identify and develop a miRNA-based prediction model for distinguishing between SCC and AC in surgical resected specimens. Surgical tissues were obtained from 128 lung cancer patients who had either a lobectomy or a pneumonectomy between March 1, 2013 and September 25, 2015 at the UMMC. Cryostat microtome and hematoxylin-eosin stained slides were prepared from the resected specimens for histopathological assessment and classified based on WHO classification of tumors of the lung [47]. Furthermore, manual macrodissection was performed on the tissues as described in our previous study [35] to ensure the presence of more than 85% cancer cells in each sample. None of the patients had received preoperative adjuvant chemotherapy or radiotherapy. 2), to confirm the prediction model for the subclassification of NSCLC in a different set of lung tumor tissue specimens, FFPE specimens were prepared from the surgical tissues of 112 lung cancer patients who had either a lobectomy or a pneumonectomy at Hebei Medical University. 3), to determine if the prediction model could be useful in bronchoscopically collected BALs for distinguishing SCC from AC, from April 1, 2010 and July 28, 2013, we prospectively recruited patients who underwent bronchoscopy because of suspicious clinical or radiological findings at the UMMC. Inclusion criteria were the followings: histological confirmed diagnosis of NSCLC with endobronchial or transbronchial biopsy, age older than 21 years and written informed consent of each patient. BAL samples were collected from the subjects as previously described [36, 48]. Briefly, flexible bronchoscopy was performed in the patients. BAL specimens were obtained from the area of the suspected lesion (as defined by endoscopy and CT scans) after the instillation of 20 cc of sterile saline and before the collection of bronchial and/or transbronchial biopsy and brushing specimens. One half of the resulting BAL fluid was centrifuged at 1,000xg for 15 min for immediate routine cytological assessments [11, 22–29, 38, 49–57]. Briefly, cytospin slides were prepared and underwent Papanicolaou staining for evaluating whether the specimens were representative of deep bronchial cells [58, 59]. To ensure the accuracy of unbiased scoring, subtypes of the lung carcinoma in the BAL materials were independently determined by two experienced cytopathologists. The selection of BAL samples for this study was undertaken on the basis of an adequate cytology preparation containing endobronchial cells and alveolar macrophages, and metaplastic or tumor cells [36]. One hundred and twenty seven specimens met these criteria and thus were used in this project.

### RNA isolation

Total RNA containing small RNA was extracted from frozen tissues and cell pellets of BALs by using a mirVana miRNA Isolation Kit (Ambion, Austin, TX, USA) as described in our previous study [20, 27, 33, 38, 60]. Four μm-thick FFPE sections were deparaffinized by using xylene, washed in ethanol, and digested with Proteinase K (Sigma-Aldrich) [31], from which total RNA was isolated using a RecoverAll Total Nucleic Acid Isolation Kit (Ambion). The purity and concentration of RNA were determined from OD260/280 readings using a dual beam UV spectrophotometer (Eppendorf AG, Hamburg, Germany). RNA integrity was determined by capillary electrophoresis using the RNA 6000 Nano Lab-on-a-Chip kit and the Bioanalyzer 2100 (Agilent Technologies, Santa Clara, CA, USA). Only RNA samples with integrity number values > 6 underwent in further analysis.

### RT-PCR

The expression levels of miRNAs in tumor tissues were analyzed by using real-time RT-PCR with Taqman miRNA assays (Applied Biosystems, Foster City, CA, USA) as previously described [27]. U6 was used as internal control gene. Furthermore, relative expression of a targeted miRNA in a given sample was computed using the equation 2−ΔCt, where ΔCt = Ct (targeted miRNA) – Ct (internal control gene, U6). Ct values were defined as the fractional cycle number, in which, the fluorescence crossed the fixed threshold. All assays were performed in triplicates. Furthermore, two interplate controls and one no-template control were carried along in each experiment. The no template control for RT was RNease free water instead of RNA sample input, and no template control for PCR was RNease free water instead of RT products input.

### Droplet digital PCR (ddPCR)

ddPCR for analysis of expression level of the miRNAs in BAL samples was performed as described in our previous work [38, 39]. Briefly, TaqMan™ reaction mix (Applied Biosystems) containing sample cDNA was partitioned into aqueous droplets in oil via the QX100 Droplet Generator (Bio-Rad, Pleasanton, CA, USA), and then transferred to a 96-well PCR plate. A two-step thermocycling protocol (95°C ×10min; 40 cycles of [94°C ×30s, 60°C ×60s], 98°C ×10 min) was undertaken in a Bio-Rad C1000 (Bio-Rad). The PCR plate was loaded on Droplet Reader (Bio-Rad), by which copy number of each miRNA per μl PCR reaction mixture was directly determined. Expression of a targeted miRNA in a given sample was calculated using the same equation as described above. However, instead of using Ct, we used copy number of gene in the equation: expression of a miRNA in a given sample = 2−Δcopy number of gene, where Δcopy number of gene = copy number of gene (targeted miRNA) – copy number of gene (U6).

### Statistical analysis

Based on one-sample with binomially distributed outcomes, we needed 45 cases from each histological type of NSCLC at 5% significant level with 80% power to discover and validate biomarkers. We computed the receiver operating characteristic (ROC) curve and used the area under the ROC curve (AUC) as an accuracy index for evaluating the diagnostic performance of the miRNAs [10]. We used stepwise logistic regression models to construct diagnostic biomarker panels, and then used the stepwise backward model selection to identify the best discriminating combinations of miRNAs for classification of SCC from AC. We employed the McNemar chi-squared test to determine the significant differences between cytology and biomarker panel. All *P* values were two-sided, with values < 0.05 considered statistically significant.
